# A Nanotube Injector for Cytoplasmic Transfer and Enhanced Mitochondrial Function

**DOI:** 10.1002/smsc.202500598

**Published:** 2026-03-17

**Authors:** Bingfu Liu, Zhuhang Dai, Bowen Zhang, Kazuhiro Oyama, Chenxi Li, Yukun Chen, Mingyin Cui, Takeo Miyake

**Affiliations:** ^1^ Graduate School of Information Waseda University Kitakyushu Fukuoka Japan

**Keywords:** adenosine triphosphate synthesis, mitochondria transfer, molecular delivery, nanotubes membrane

## Abstract

The intercellular transportation of molecules is crucial for regulating cell communication and function. However, the existing techniques for molecule transfer across cell barriers often cause cellular damage or have low transfer efficiencies. To address these limitations, this study proposes an innovative nanotube membrane‐based injector (nanoinjector) system capable of extracting diverse cytoplasmic molecules from source cells and transferring them to target cells. The developed system demonstrates high efficiency, with over 95% viability and 90% transfer efficiency. Additionally, it enables mitochondrial transfer, which enhances cellular adenosine triphosphate (ATP) production by up to 25% within 24 h. This study explores the impact of intracellular content transport, enabled by this new tool, on cellular activities, with promising implications for cell surgery and therapy.

## Graphical abstract

We propose a gold nanotube membrane‐based injector (nanoinjector) system for efficient intracellular molecule transfer, achieving over 95% cell viability and 90% transfer efficiency. The system enables mitochondrial transfer, enhancing adenosine triphosphate production by 25% for 24 h. This approach holds promise for improving cellular therapies and cell surgery by facilitating targeted content transport without significant cellular damage.

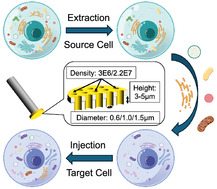

## 1 Introduction

Intercellular cytoplasm transfer represents a paradigm shift in our understanding of cell communication. This process involves the exchange of cytoplasm components such as proteins [1, 2], DNA [3], RNA molecules [4], and even organelles between adjacent cells, connecting with tunneling lipid nanotubes. Notably, one crucial organelle in this exchange is the mitochondrion [5], which serves as the powerhouse of cells, driving energy production and cellular metabolism [6]. For example, leukemia cells [7] can survive by receiving mitochondria from surrounding cells in the body. Cancer cells exploit intercellular mitochondrial transfer to maintain elevated metabolic demands and promote drug resistance [8]. Similarly, mitochondrial transfer from adipose stem cells to dermal fibroblasts in subcutaneous tissues has been reported to improve the aging process of fibroblasts [9]. Thus, transferring functional molecules into different cell types is important for enhancing cellular functionality [10, 11].

To technically achieve such cytoplasm transport, two steps are required: extraction of cytoplasm from source cells and delivery of the extracted cytoplasm into target cells. Cell lysis with detergents or enzymes [12] is a common technique for extracting intracellular components, but compromises cellular functions during/after the process. Utilizing the mechanical energy of ultrasound [13] to rupture the cell membrane and release cytoplasm is a highly effective method, but careful control of the ultrasound intensity is required to avoid damaging target molecules. Moreover, the delivery of target molecules into cells can be classified into chemical (lipid‐ and viral vector‐based [14]) and physical (electroporation [15] and micro/nanoinjection) methods [16]. However, crucial issues persist: lipid‐based nanocarriers are limited to small molecules [17], viral vectors generally require long processing times and costs [18], electroporation [19] can damage cells [20], and micro/nano‐injection has low efficiencies [21]. The only artificial method reported to achieve intercellular cytoplasm transport is a microfluidic atomic force microscope (AFM). This technique can apply a negative pressure to the AFM probe to generate drops with accurate volumes in picoliters that can be sucked through a triangular aperture one by one; thus, this technique can be utilized for single‐cell analysis [22, 23, 24, 25]. Therefore, a cytoplasm transfer technique requires accurate control of the extraction and delivery of intracellular molecules while maintaining operability and scalability to handle large numbers of cell experiments.

**FIGURE 1:**
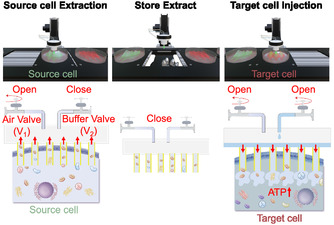
Schematic of the cytoplasmic transfer system using a nanotube‐based injector. The system extracts cytoplasmic molecules from source cells (extraction), store the extracted molecules within the nanotubes by controlling the air valve (*V*_1_; store), and subsequently delivers the molecules into target cells by opening both *V*_1_ and *V*_2_ valves while adding buffer solution (injection).

Here, we propose a nanoinjector system to facilitate cytoplasmic transfusion, encompassing not only the soluble components but also the transfer of essential organelles such as mitochondria. The system can insert nanotubes (NTs) into cells and extract and/or inject the molecules from/into the cells by controlling the inner pressure of the NT stamp (Figure 1). We propose a novel cytoplasm transfer system for facilitating direct transfer between cells, thereby ensuring high cell activity and excellent transfer efficiency.

## 2 Results and Discussion

### 2.1 Intracellular Extraction With a Nanoinjector

**FIGURE 2:**
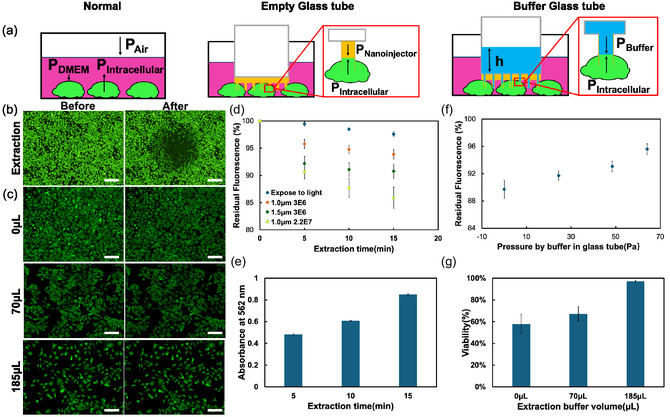
Cytoplasm extraction from source cells: (a) Schematic of extraction using a nanotube stamp. “Normal” represents no nanotube use; “Empty Glass tube” mean the state of glass tube connected to nanotube stamp during cell insertion and “Buffer Glass tube” means a glass tube with a buffer solution added before cell insertion. (b) Fluorescence images of calcein‐stained HeLa cells before and after the extraction for 15 min with a nanotube stamp using an empty glass tube (no additional buffer solution). (c) Fluorescence images of calcein‐stained HeLa cells before and after the extraction for 5 min with a nanotube stamp adding buffer solutions of 0, 70, and 185 μL. (d) Residual fluorescence values of calcein‐stained HeLa cells at the different extraction times using different nanotube stamps of 1.0 μm, 3E6 (orange), 1.5 μm, 3E6 (green), and 1.0 μm, 2.2E7 (yellow). Control experiments (blue) were conducted without the insertion. (e) Absorbance values at 562 nm at different extraction times using BCA method. (f) Residual fluorescence values at different extraction times using a nanotube stamp of 1.0 μm, 2.2E7 under different pressures. (g) HeLa cell viability after 24 hr incubation with different buffer volumes in the nanotube stamp. Values are mean ± SD (*n* = 3). Scale bars are (b) 50 and (c) 200 μm.

To achieve simultaneous extraction of cytoplasm from source cells and its subsequent injection into target cells, we designed a nanoinjector system. The gold NT stamp, as described in previous literature [26, 27, 28, 29], consists of a gold NT membrane and a glass tube (Figure S1). The principle behind extracting intracellular fluid is straightforward. When an empty NT stamp is inserted into a cell, the intracellular fluid flows into the stamp through the NTs if the pressure inside the stamp tube (*P*_Nanoinjector_ = *P*_Air_) is lower than the cell's internal pressure (*P*_Intracellular_) (Figure 2a). To experimentally verify the above, HeLa cells were stained with Calcein‐AM, and the intensities of the stained cells were measured before and after the insertion of the NT stamp at different times. From the fluorescence images in Figure 2b,c, the average intensities before and after the insertion were denoted as *I*_before_ and *I*_after_, and the average intensities of the background were *B*_before_ and *B*_after_, respectively. Fluorescence intensity was quantified from 50 cells (*n* = 50) per condition. The mean gray value was measured for each cell after background subtraction. Finally, the residual values (%) were calculated by the following equation,

(1) $$R\;\left(\%\right)=\frac{I_{\text{after}}-B_{\text{after}}}{I_{\text{before}}-B_{\text{before}}}\times 100\text{\textbackslash{}\%}$$

The *R*‐value without insertion was 97.6 ± 0.4% at 15 min, indicating that photobleaching during the measurements was negligible (Figure 2d). In contrast, for insertion using a nanoinjector with a NT diameter of 1 μm and a tube density of 3 × 10^6^ cm^−2^ (1.0 μm, 3E6), the *R*‐value decreased to 93.9 ± 0.9% at 15 min. Furthermore, when we increased the NT diameter and density, the *R* values decreased to 90.8 ± 1.3% at 1.5 μm, 3 × 10^6^ cm^−2^ (1.5 μm, 3E6) and 85.9 ± 2.0% at 1.0 μm, 2.2 × 10^7^ cm^−2^ (1.0 μm, 2.2E7). Given that the average area of the adhesive HeLa cells was 400 ± 100 μm^2^, ≈12 ± 8 NTs were inserted into each cell at a density of 3E6, and 80 ± 5 NTs at a density of 2.2E7.

To isolate the specific effect of intracellular fluid extraction on the *R*‐value and rule out confounding variables such as pH variation, we performed control experiments using the pH‐sensitive dye 2′, 7′‐bis‐(2‐carboxyethyl)‐5‐(and‐6)‐carboxyfluorescein (BCECF‐AM). Ratiometric fluorescence analysis revealed no significant changes in intracellular pH following NTs insertion across all tested time points (Figure S2). Furthermore, we quantified the total protein in the extracts by using the Bicinchoninic Acid Assay (BCA) method, relying on absorbance measurements without employing fluorescent dyes. By systematically comparing the detection results of samples with different extraction times, we found that the characteristic absorbance value of the sample at 562 nm increased significantly with the extension of extraction time (Figure 2e).

Improved control of the extraction flow from the source cell to the NT stamp was achieved by controlling the *P*_Nanoinjector_ pressure (Figure 2a,f). When buffer solution was added to the NT stamp, *P*_Nanoinjector_ increased by the liquid pressure *P*_Buffer_, which is defined as,

(2) $$P_{\text{Buffer}}=\rho gh$$

where, ρ is the liquid density (tris buffer = 1000 kg m^−3^), g is gravitational acceleration (9.8 N kg^−1^), and h is the height of the buffer solution in the NT stamp. For 5 min extraction, without buffer (0 μL), the *R*‐value in the NT stamp was 89.7 ± 1.3%. This increased to 91.7 ± 0.7% with 70 μL (*P*_Buffer_ = 24.4 Pa), 93.1 ± 0.8% with 140 μL (*P*_Buffer_ = 48.41 Pa), and 95.6 ± 0.8% with 185 μL (*P*_Buffer_ = 64.09 Pa). Adding more buffer solution did not increase the *R*‐value over 96.5% due to the dilution of Calcein inside the cells. Adding 70 μL of buffer solution to a glass tube with an inner diameter of 6 mm for the NT membrane resulted in *h* = 2.49 mm, which was equal to that of the cell culture medium, and the extraction flow from the source cells could not be suppressed. To suppress the extraction flow, we found that > 64 Pa at *h* = 6.54 mm (adding 185 μL) was required.

Regarding cell viability, after cells were extracted using a NT stamp of 1.0 μm, 2.2E7 from 5 min to 15 min, cell death was monitored after 24 hr (Figure S3). The cell viability was 57.91 ± 8.67% for 5 min, 41.84 ± 5.29% for 10 min, and 18.34 ± 6.41% for 15 min, indicating that a longer extraction time increases damage to the cells. Using the same 15 min extraction time with different NT stamps (Figure S4), the cell viability was 38.01 ± 6.34% for 1.0 μm, 3E6, 31.01 ± 5.95% for 1.5 μm, 3E6, and 18.34 ± 6.41% for 1.0 μm, 2.2E7. Similarly, a larger diameter and a higher density of the NT increase the damage to the cell. After adding the buffer to the NT stamp and extracting it after 5 min (Figure 2g), the viability increased to 57.91 ± 8.67% for 0 μL, 67.08 ± 6.72% for 70 μL, and 97.02 ± 1.02% for 185 μL. When the downward pressure exerted by the buffer is approximately equal to the internal pressure of the cell, free diffusion occurs within the NTs. This allows the intracellular fluid to exchange with the buffer solution rather than be extracted, which maximizes the preservation of cell integrity within a reasonable timeframe, reduces cell damage, and prevents large‐scale cell death. These findings indicate that our nanoinjector system can effectively extract molecules from source cells while allowing precise control of the extraction flow, achieved by adjusting NT diameters and injecting additional buffer solutions.

### 2.2 Transfer of the Extracted Molecules With the Nanoinjector

**FIGURE 3:**
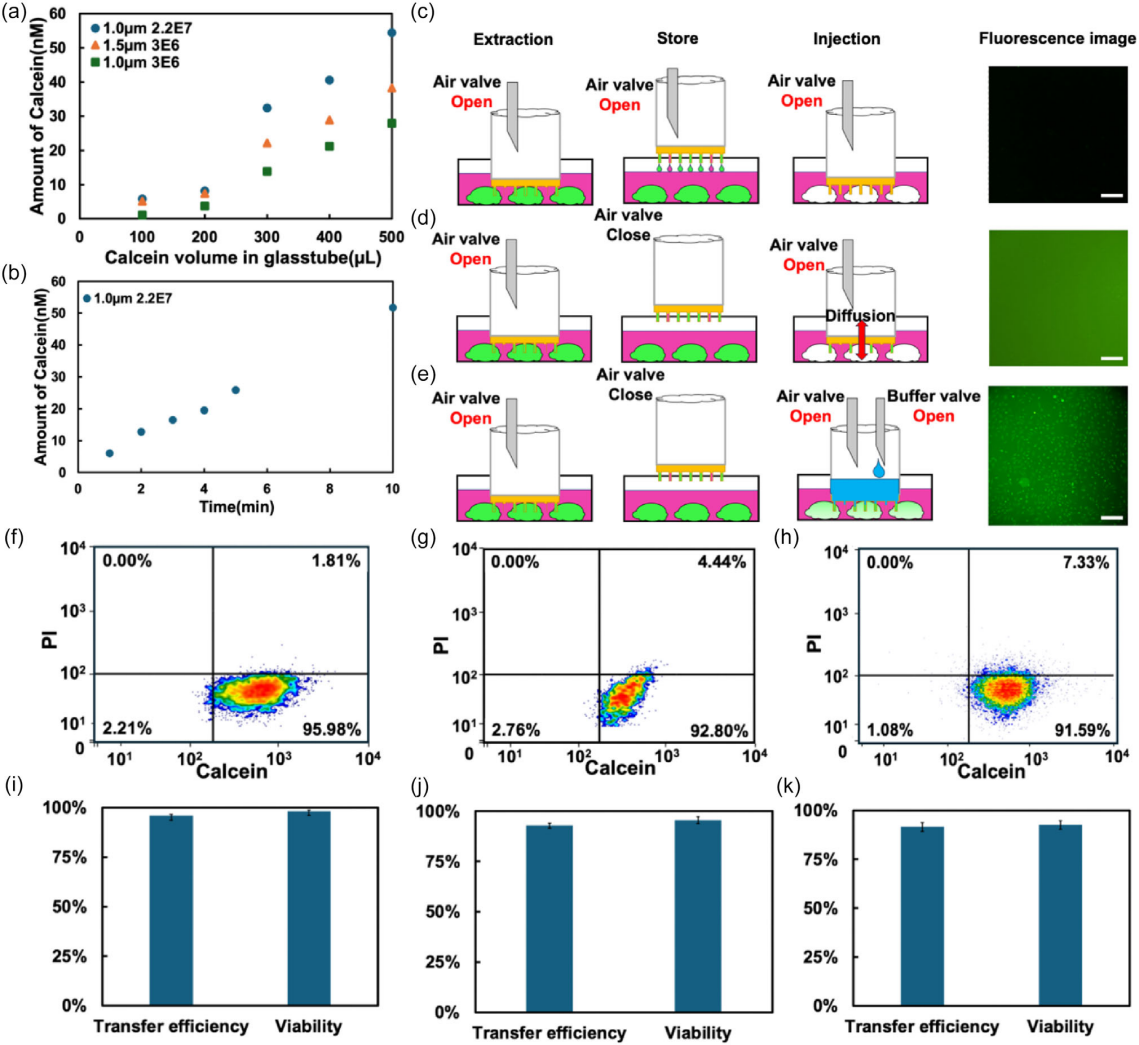
Calcein transfer from source to target cells. (a) Amount of transported calcein using 1.0 μm, 3E6, 1.5 μm, 3E6, and 1.0 μm, 2.2E7 nanotube stamps with different volumes of calcein solution in the source chamber. (b) Amount of transported calcein at different times using 300 μL of calcein. (c–e) Schematic representation of the cytoplasm transfer process with (c) *V*_1_ constantly open, extract cannot be stored, thus injection is not successful; (d) Closing *V*_1_ after extraction allows the processed extract to be temporarily stored in the nanotubes. Opening *V*_1_ during injection, closing *V*_2_ cannot provide enough pressure to allow the extract to enter the target cells, resulting in only minimal diffusion; (e) Opening *V*_1_ and *V*_2_ during injection buffer solution can provide sufficient pressure for the extract to smoothly enter the target cells. (f–h) Flow cytometry analysis of cells treated with nanotube stamps with glass tube diameters of (f) 6 mm, (g) 8 mm, and (h) 18 mm. (i–k) Transfer efficiencies and cell viabilities obtained from the data in f–h for: (i) 6 mm, (j) 8 mm, and (k) 18 mm glass tube diameters with a 1.0 μm, 2.2E7 nanotube membrane. Scale bar is 200 μm. Values are mean ± SD (*n* = 3).

To calibrate the transfer of the extracted intracellular solution to target cells, we first investigated the injection flux of calcein from the NT stamp. We prepared a NT stamp with varying volumes of 1.6 mM calcein dye to control the inner pressure (*P*_Nanoinjector_) and submerged it into micro‐wells to a depth of around 2.5 mm below the water surface. The concentration of calcein released from the tips of the NTs was measured using a fluorescence microplate reader (Figure 3a). The results showed a significant difference in the amount of calcein released when adding volumes in the range from < 200 μL to > 300 μL. This suggests two mechanisms of molecular delivery: one is the diffusive release of calcein (for volumes ≤200 μL), and the other is utilizing the water pressure of the additional buffer solution to accelerate the flow of the target molecules into the chamber (for volumes ≥300 μL). Interestingly, the amount of released calcein increased 1.4‐fold when the diameter of the NTs was increased to 1.5 μm, 3E6, and 2.1‐fold when the density of the NTs was increased to 1.0 μm, 2.2E7. When we used a nanoinjector with 1.0 μm, 2.2E7 and loaded 300 μL of calcein solution onto the NT stamp, the calcein flux was 5.07 nmol s^−1^ cm^−2^ (Figure 3b), which closely matches our previous studies [26, 27].

To transport intracellular fluid extracted from source to target cells, we developed a NT stamp with a dual‐valve system: one valve to regulate internal pressure in the glass tube and another one to add buffer solution. As shown in Figure 3c–e, attempts to deliver extracted fluid with the air valve (V_1_) open resulted in leakage, preventing successful transfer (Figure 3c). When V_1_ was closed after extraction, the stamp could contact the target cell with the residual solution, but delivery into the cell remained unsuccessful. Similarly, opening V_1_ during stamping also failed to achieve transport (Figure 3d). These results underscore the necessity of precisely controlling the internal pressure within the NT stamp, as indicated in Figures 2 and 3a. To overcome this limitation, we opened V_1_ and introduced 300 μL of buffer solution during delivery, which enabled successful transfer of calcein dye from source to target cells, confirmed by fluorescence imaging (Figure 3e).

To determine the transfer efficiency of our system, we used NT stamps with different glass tube diameters of 6, 8, and 18 mm to transport calcein‐containing solutions from source to target cells (Figure S5) and measured the number of cells with and without calcein transport using flow cytometry (FCM) (Figure 3f–h). The delivery efficiencies were 95.98 ± 0.69% (18343 calcein‐delivered cells, 768 non‐delivered cells), 92.80 ± 1.25% (45560 calcein‐delivered cells, 3534 non‐delivered cells), and 91.59 ± 2.25% (91590 calcein‐delivered cells, 1080 non‐delivered cells) with 6, 8, and 18 mm glass tube diameters, respectively. Additionally, we evaluated the cell viability by staining all cells with propidium iodide (PI). We obtained a cell viability of 98.19 ± 0.75%, 95.56 ± 1.64%, and 92.67 ± 2.14% with 6, 8, and 18 mm glass tube diameters, respectively (Figure 3i–k).

### 2.3 Transfer of the Extracted Molecules to Different Types of Cells

**FIGURE 4:**
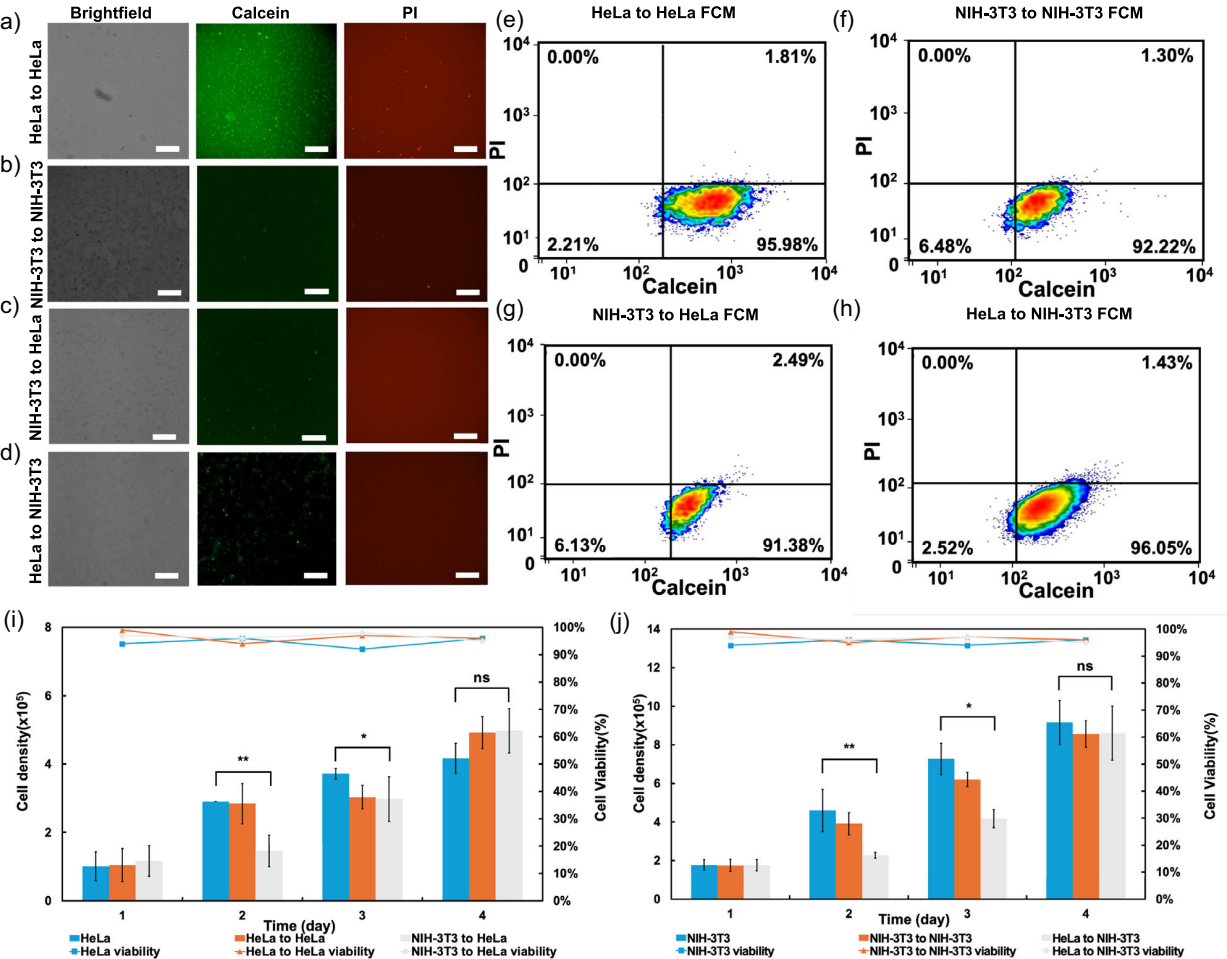
Cytoplasm transfer from source to target cells. Optical and fluorescence images of (a) HeLa cells after transfer from HeLa source cells, (b) NIH‐3T3 cells after transfer from NIH‐3T3 source cells, (c) HeLa cells after transfer from NIH‐3T3 source cells, (d) NIH‐3T3 cells after transfer from HeLa source cells. Fluorescence images in each case indicate Calcein (green) and PI (red). Flow cytometry analysis after transferring e) from HeLa to HeLa cells, (f) from NIH‐3T3 to NIH‐3T3 cells, (g) from NIH‐3T3 to HeLa cells, (h) from HeLa to NIH‐3T3 cells. (i–j) Cell densities and viabilities after transfer at different days: (i) HeLa cell and (j) NIH‐3T3 cell. Scale bar is 200 μm. Values are mean ± SD (*n* = 3). Unpaired two‐tailed Student's t‐tests were used. Significance level was implied by *, **, ***, and ns for *p* < 0.05, *p* < 0.01, *p* < 0.001, and no significance, respectively.

Using our system, we transferred intracellular fluid between HeLa and NIH‐3T3 cells, as well as cells of the same type (HeLa–HeLa, NIH‐3T3‐NIH‐3T3) (Figure 4). Similar to Figure 3f–h, delivery efficiencies and viabilities were assessed by flow cytometry, quantifying cells positive for calcein dye and cells stained with PI. The results showed a delivery efficiencies and viabilities of 95.98 ± 0.69% and 98.19 ± 0.75% for HeLa–HeLa (Figure 4a,e), 92.22 ± 2.34% and 98.70 ± 0.34% for NIH‐3T3‐NIH‐3T3 (Figure 4b,f), 91.38 ± 1.34% and 97.51 ± 0.76% for NIH‐3T3‐HeLa (Figure 4c,g), and 96.05 ± 1.25% and 98.57 ± 0.23% for HeLa‐NIH‐3T3 (Figure 4d,h), respectively.

Furthermore, we evaluated cell densities with and without transportation. After 4 days of incubation, the transferred cells displayed normal growth. This may be attributed to the relatively small amount of cytoplasmic solute transferred. Notably, as a control experiment, when intracellular fluid was transferred between cells of the same species (HeLa to HeLa and NIH‐3T3 to NIH‐3T3), there were almost no differences in growth rates compared to the original cells. However, growth rates significantly declined by the second day when we transported intracellular fluids between different types of cells (HeLa to NIH‐3T3, NIH‐3T3 to HeLa), as shown in Figure 4i,j. For HeLa cells, while initial cell numbers were comparable on day 1, a significant suppression of proliferation was observed in the NIH‐3T3 to HeLa group on day 2 (2.90 ± 0.01 vs. 1.41 ± 0.46; *p* < 0.01). This suppressive effect persisted but diminished by day 3 (3.72 ± 0.15 vs. 2.98 ± 0.66; *p* < 0.05). On day 4, the transfer group cell numbers recovered and increased, showing no significant difference compared to the HeLa group (*p* > 0.1). For NIH‐3T3 cells, while the initial cell numbers were comparable on day 1, a significant suppression of proliferation was observed in the HeLa to NIH‐3T3 group on day 2 (4.61 ± 1.09 vs. 2.27 ± 0.15; *p* < 0.01). This suppressive effect diminished by day 3 (7.27 ± 0.81 vs. 4.17 ± 0.47; *p* < 0.05). On day 4, the transfer group cell count recovered and increased, showing no significant differences compared to the NIH‐3T3 group (*p* > 0.1). The transient inhibition of cell proliferation by foreign cytoplasm may be due to the cytoplasm's inherent “non‐self” recognition and clearance. Signaling molecules introduced by foreign cytoplasm disrupt signal transduction, leading to cell cycle arrest. As the foreign components are gradually cleared, cells resume normal metabolism and proliferation, sometimes even exhibiting a compensatory growth rate exceeding that of the control. Regarding the cell viability curve, all cells showed high viability of >90% during the monitored time span of 4 days.

### 2.4 Mitochondria Transfer With the Nanoinjector

**FIGURE 5:**
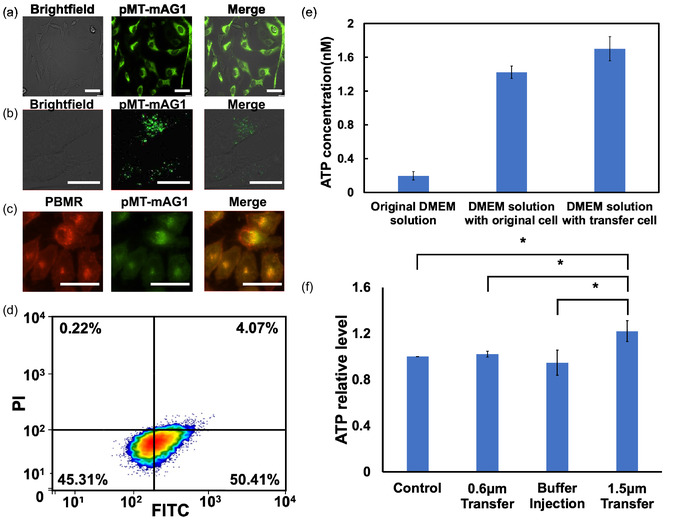
Mitochondria transfer between HeLa cells: optical and fluorescence images of (a) HeLa cells, mitochondria stained by pMT‐mAG1 plasmid. (b,c) mitochondria transferred between HeLa cells. Fluorescence images indicate pMT‐mAG1 (green) and PlasMem Bright Red (red). (d) Flow cytometry analysis after transferring mitochondria. ATP concentration test after mitochondria transfer: (e) culture solution test with or without mitochondria transfer; (f) intracellular ATP concentration growth rates with or without mitochondria transportation compared to the original cells. Scale bar is 50 μm. Values are mean ± SD (*n* = 3). One‐way ANOVA with Dunnett's post hoc test and Holm‐adjusted planned comparisons were applied. Significance level was implied by *, **, ***, and ns for *p* < 0.05, *p* < 0.01, *p* < 0.001, and no significance, respectively.

Following the transportation of intracellular fluid using our system, we evaluated whether it results in changes in cellular functions. We focused on mitochondrial transfer and quantitatively assessed the amount of ATP synthesis in the target cells. The mitochondria in the HeLa source cells were stained with mitochondria‐targeted monomeric Azami‐Green (pMT‐Mag1) plasmid (Figure 5a). After transport using our nanoinjector system, fluorescence from the labeled mitochondria was observed in the target cells (Figure 5b). We confirmed that fluorescence‐labeled mitochondria were inside the cell using confocal microscope observation, labeling the membrane with PlasMem Bright Red (PMBR) (Figure 5c). Because PMBR can penetrate into cells and mark some organelle membranes, overlaying green fluorescence from transferred mitochondria with red fluorescence from target cell membranes clearly demonstrated our successful mitochondrial transfer. On average, ≈35 ± 10 mitochondria were delivered per cell, and the target cell viability remained high (95.72%), as measured by FCM (Figure 5d).

ATP production in target cells was quantified before and after mitochondrial transfer. As a control, the first ATP released into the culture medium was measured. As shown in Figure 5e, the ATP released from the transferred target cells to the culture medium (1.70 nM) was higher than that without the mitochondria transferred (1.42 nM), while the original culture medium without cell culture was at 0.20 nM. To evaluate ATP synthesis inside the cells, we conducted an experiment where cells were cultured for 1 day after the NT stamp insertion, including 0.6 μm, 4 × 10^7^ cm^−2^ (0.6 μm, 4E7) transfer and 1.5 μm, 3E6, for only the buffer solution and the extracted mitochondrial solution (Figure 5f). As hypothesized, intracellular ATP levels were quantified and normalized to untreated control cells. One‐way ANOVA revealed a significant difference in ATP levels among the four groups. Notably, for the same 1.5 μm NT stamp, transfer resulted in significantly higher ATP levels compared with buffer injection (*p* < 0.05). Furthermore, ATP levels in the 1.5 μm NT transfer were higher than those in the 0.6 μm NT transfer (*p* < 0.05). The diameter of 0.6 μm is too small and not well‐suited to the size of mitochondria. Without additional negative pressure, it would be difficult to extract, and it may only transfer some ATP that exists in the cytoplasm. Buffer solution injection may dilute the cytoplasm, thus probably decreasing the measured results.

These results indicated that our system could transfer the cytoplasm, including mitochondria, from source to target cells, and then improve their ability to produce ATP molecules inside the target cells.

## 3 Conclusion

In conclusion, we developed a nanotube membrane‐based injector system for cytoplasmic transfer. This method enables the simultaneous extraction, preservation, and delivery of cytoplasmic solutes through controlling pressure differentials across the cell membrane. The system maintains high cell viability (≈95%) and achieves a transfer efficiency of ≈90%. Moreover, it supports intercellular mitochondrial transfer, with a ≈25% increase in ATP concentration compared to a control without transfer, confirming the retained functionality of transferred mitochondria. Overall, this approach holds strong potential for facilitating molecular exchange between cells and for advancing the analysis of intracellular composition.

## 4 Experimental Section

### 4.1 Materials and Instruments

Calcein‐AM (Thermo Fisher Scientific, Catalog Number:C3099), 2′,7′‐bis‐(2‐carboxyethyl)‐5‐(and‐6)‐carboxyfluorescein (BCECF‐AM, DOJINDO, Catalog Number: B262), propidium iodide (PI, DOJINDO, Catalog Number: P378), Mitochondria‐targeted monomeric Azami‐Green plasmid (pMT‐Mag1, MBL International Corporation, Catalog Number: AM‐V0201M), PlasMem Bright Red (PMBR, DOJINDO, Catalog Number: P505), and ATP Bioluminescence Assay Kit CLS II (Sigma–Aldrich, Catalog Number: 12352204).

Scanning electron microscope (SEM) images were acquired using a JEOL SEM JSM‐IT200 (JEOL, Japan). Fluorescence images were acquired using an Olympus IX83 inverted fluorescence microscope equipped with a confocal laser scanning system (FV3000, Olympus, Japan). Flow cytometry data were collected using a Sysmex RF‐500 (Sysmex Corporation, Japan).

### 4.2 Cell Culture

For cytosol experiments, HeLa (RIKEN BioResource Research Center, Catalog Number: RCB0007) and NIH‐3T3 (RIKEN BioResource Research Center, Catalog Number: RCB2767) cells were selected as adherent cell models. These cells were cultured in Dulbecco's Modified Eagle medium (DMEM, Thermo Fisher Scientific, Catalog Number: 12800017) supplemented with 10% fetal bovine serum (Thermo Fisher Scientific, Catalog Number: 26140079) and 1% penicillin (Sigma–Aldrich, Catalog Number: P7794)–streptomycin (Sigma–Aldrich, Catalog Number: S6501) to support growth and survival. To prepare a cell suspension, 0.5% trypsin (Sigma–Aldrich, Catalog Number: T4049) was used. The cells were maintained in a 5% CO_2_ incubator at 37°C to ensure optimal growth conditions. For the mitochondrial transfer experiments, HeLa cells transfected with the pMT‐mAG1 plasmid were used as the source. All cell types used were from generations 6–17 and ensured that the number of passages in each experiment was consistent.

### 4.3 BCA Method for Detecting Proteins in Extracts

Before the extraction experiment, polydimethylsiloxane (PDMS) donuts were used to select the injection area, and cultured HeLa cells were inside. Then, PDMS donut was removed, and cells were washed three times with phosphate‐buffered saline (PBS) to ensure the removal of serum‐provided proteins from the culture medium. The extraction experiment was conducted immediately. After extraction, 100 μL of PBS was drawn into a syringe, and the extract was expelled. Simultaneously, 100 μL of PBS held with the cells was used as a control group. Using the TaKaRa BCA Protein Assay Kit (TaKaRa, Catalog number: T9300A), 100 μL of working solution was added, and the mixture was incubated at 60°C for 1 h. The absorbance was measured at 562 nm using a nanophotometer (Wakebtech, np80) and calibrated at 650 nm.

### 4.4 DNA Plasmid Transfection

The day before transfection, HeLa cells were seeded at a density of 4 × 10^5^ cells per dish in a 35 mm petri dish and incubated overnight at 37°C, 5% CO_2_ in a cell culture incubator. pMT‐Mag1 is a plasmid that labels mitochondria green. On the day of transfection, a transfection mixture was prepared by combining 1.5 μg DNA, 100 μL DMEM without serum, proteins, or antibiotics, and 12 μL polyfect transfection reagent (QIAGEN, Catalog number:301105) in a small tube. The mixture was allowed to stand for 5−10 min to facilitate complex formation. The culture medium from the original culture dish was removed, and the cells were washed with 2 mL PBS. Subsequently, the transfection mixture and DMEM (with serum, proteins, or antibiotics) were added to the culture dish and mixed thoroughly for co‐cultivation. After 2 days, the cells were passaged at a density of 3.2 × 10^5^ cells per well, and geneticin (G418) was added to each petri dish at a concentration of 1.2 mg mL^−1^ for culturing. Precisely 1 week later, the transfected cells were seeded into 48‐well plates at a density of 2.5 × 10^2^ cells per well. The culture medium was regularly replaced, and G418 was used for iterative selection to isolate individual cells exhibiting significant mitochondrial fluorescence for further propagation. Finally, the selected cells were preserved in a liquid nitrogen‐filled tank.

### 4.5 Statistical Analysis

The experimental data were organized and analyzed by using Microsoft Excel, ImageJ software version 1.53t, and GraphPad Prism 10.6.0. The data were expressed as mean ± standard deviation (SD). The residual values measurement, transfer between different cells, cell viability, growth rate, and ATP concentration experiments comprised at least three independent experimental batches performed under identical conditions (*n* ≥ 3). For cell growth rate experiment, based on an a priori hypothesis, statistical comparisons were performed only between the untreated control group and different types of cell transfer at each time point using unpaired two‐tailed Student's t‐tests. Same type of cell transfer was included for reference but was not subjected to statistical comparison. For ATP level experiment, statistical analysis was performed using one‐way ANOVA followed by Dunnett's test for comparisons versus control. Planned pairwise comparisons (1.5 μm transfer vs. 1.5 μm buffer injection and 1.5 μm transfer vs. 0.6 μm transfer) were conducted with Holm‐adjusted *p* values. In all cases, significance levels (*p* values) were indicated with asterisks in each figure (*p < 0.05, **p < 0.01, ***p < 0.001, and *p* > 0.05 as ns).

## Supporting Information

Additional supporting information can be found online in the Supporting Information section. The supporting information is attached. **Supporting Fig. S1:** Scanning electron microscopy images of the structure of the nanotube membrane at different diameters and densities: a) 1.0 μm, 3E6, the hollow needle height was approximately 4.8‐5.2 μm; b) 1.5 μm, 3E6, the hollow needle height was approximately 3.6‐4.1 μm; c) 1.0 μm, 2.2E7, the hollow needle height was approximately 4.8‐5.2 μm. d) A physical assembly diagram of the nanoinjector comprising a gold nanotube membrane and a glass tube. **Supporting Fig. S2:** pH test for nanotube stamp: a) Fluorescence images at different insertion times under excitation at 445 nm and 485 nm. b) Average fluorescence intensity at different insertion times and the ratio of fluorescence intensity (F_485_/F_445_). Scale bar is 200 μm. **Supporting Fig. S3:** Optical and propidium iodide (PI) fluorescence (red) images of HeLa cells after 24 hr incubation at different extraction times using a nanotube stamp of 1.0 μm, 2.2E7: a) 5 min, b) 10 min, and c) 15 min. d) HeLa cell viability after 24 hr incubation with different extraction times using a nanotube stamp of 1.0 μm 2.2E7. Scale bar is 500 μm. **Supporting Fig. S4:** Optical and propidium iodide (PI) fluorescence (red) images of HeLa cells after 24 hr incubation at 15 min extraction time with different nanotube stamps: a) 1.0 μm, 3E6, b) 1.5 μm, 3E6, and c) 1.0 μm, 2.2E7. d) HeLa cell viability after 24 hr incubation with the different nanotube stamps at 15 min extraction time. Scale bar is 500 μm. **Supporting Fig. S5:** Images of cultured cells using polydimethylsiloxane (PDMS) donuts and nanotube stamps of diameters a, b) 6 mm, c, d) 8 mm, and e, f) 18 mm. Assembled image (left) and fluorescence image (right). Images of the 6 mm nanotube stamp: a) extraction, b) injection; images of the 8 mm nanotube stamp: c) extraction, d) injection, and images of the 18 mm nanotube stamp: e) extraction, f) injection.

## Conflicts of Interest

The authors declare no conflicts of interest.

## Supporting information

Supplementary Material

## Data Availability

The data that support the findings of this study are available from the corresponding author upon reasonable request.
