# Supplementary material for: A Nanotube Injector for Cytoplasmic Transfer and Enhanced Mitochondrial Function
Source: Small Sci. 2026 Mar 17;6(3):e202500598. doi: 10.1002/smsc.202500598 (PMC13097451; doi:10.1002/smsc.202500598)
Supplement: Supplementary file 1 — Supplementary Material [file SMSC-6-e202500598-s001.pdf]

Supporting information

A Nanotube Injector for Cytoplasmic Transfer and Enhanced Mitochondrial Function

*Bingfu Liu, Zhuhang Dai, Bowen Zhang, Kazuhiro Oyama, Chenxi Li, Yukun Chen, Mingyin Cui, Takeo Miyake**

Nanotube membrane-based injector

Electroless gold plating was carried out to create a tin-palladium metal catalyst layer on the surface of tracked-etched polycarbonate (TEPC). The membrane was then immersed in a gold-plating solution to produce the Au/TEPC membrane. Wet etching was performed on the surface of the membrane, and the top surface was subjected to an aqua regia reaction to remove the Au nanolayer. Subsequently, O_2 plasma was used for dry etching to expose the gold nanotubes (NTs). Next, the Au/TEPC membrane cut to a suitable size and attached to one side of the glass tube. The other side was sealed with waterproof glue to create a chamber. After assembling the chamber, injection and wing needles were inserted into it. These needles were connected to the outer glass tube, and pressure could be adjusted to facilitate the extraction, preservation, and transport of cytoplasm solution.

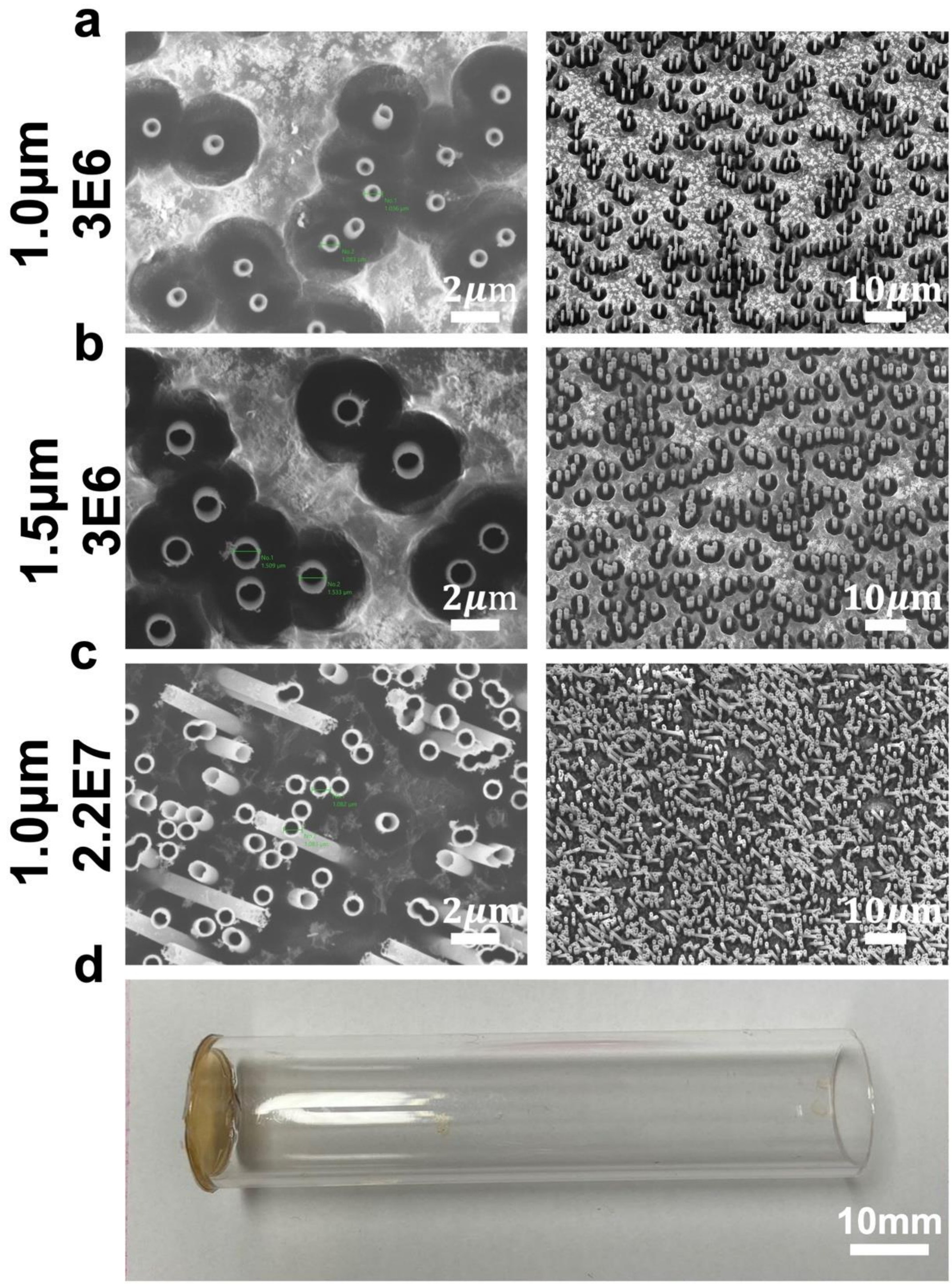

Figure S1. Scanning electron microscopy images of the structure of the nanotube membrane at different diameters and densities: a) 1.0 μm, 3E6, the hollow needle height was approximately 4.8-5.2 μm; b) 1.5 μm, 3E6, the hollow needle height was approximately 3.6-4.1 μm; c) 1.0 μm, 2.2E7, the hollow needle height was

approximately 4.8-5.2 μm. d) A physical assembly diagram of the nanoinjector comprising a gold nanotube membrane and a glass tube

pH test for nanotube stamp

To verify whether NT stamp insertion alters intracellular pH, we added BCECF-AM, a pH-sensitive fluorescent dye, to the cells before the experiment and recorded the initial fluorescence intensity. Fluorescence intensities were also recorded at 5, 10, and 15 min after insertion. pH changes were reflected by measuring the ratio of fluorescence intensity (F_{485}/F_{445}) between pH-sensitive 485 nm excitation light and pH-insensitive 445 nm excitation light.

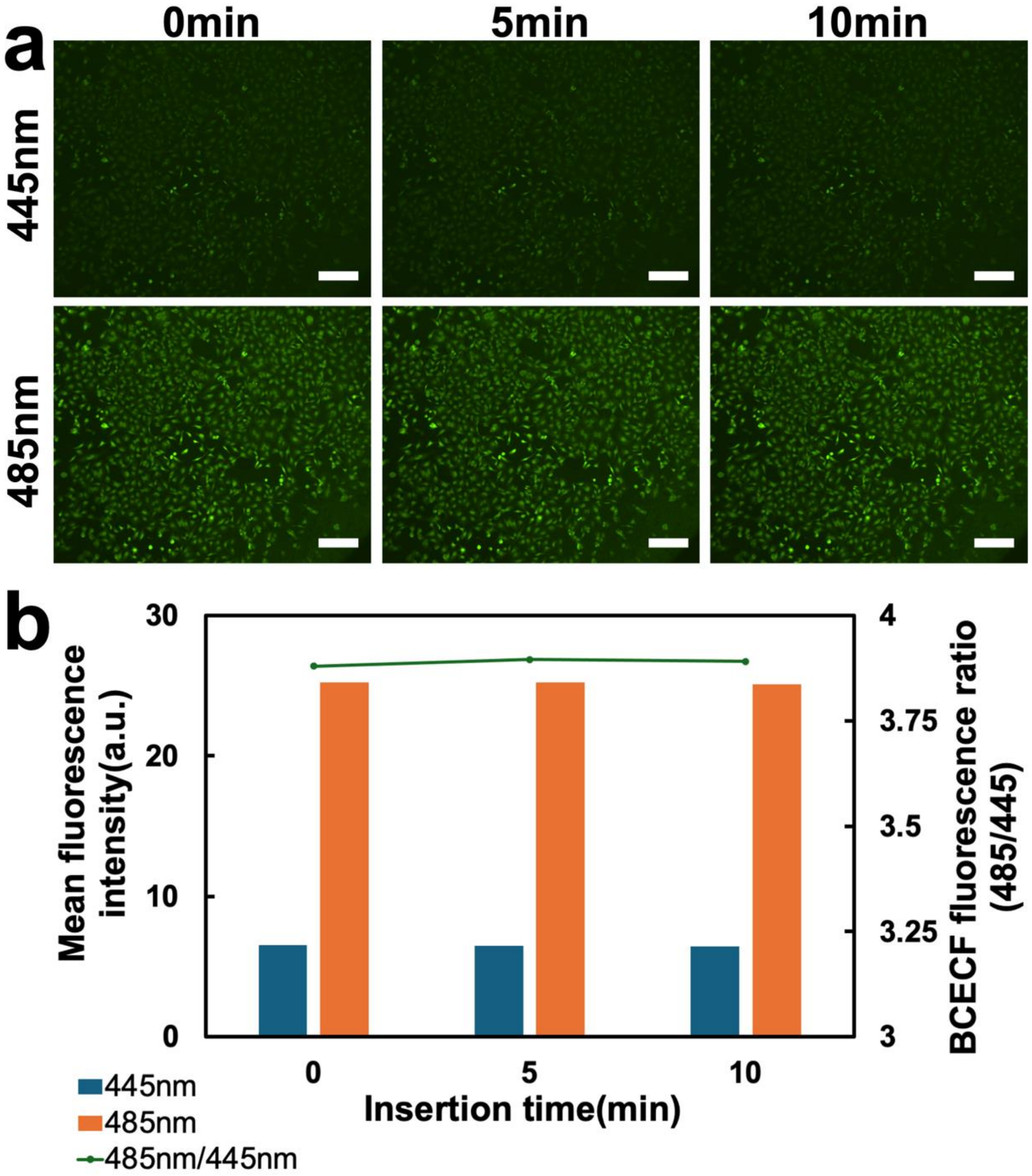

Figure S2. pH test for nanotube stamp: a) Fluorescence images at different insertion times under excitation at 445 nm and 485 nm. b) Average fluorescence intensity at different insertion times and the ratio of fluorescence intensity (F_{485}/F_{445}). Scale bar is 200 μm.

Viability for extraction

We used a NT stamp of 1.0 μm, 2.2E7 without adding any buffer and conducted the extraction with HeLa cells for 5, 10, and 15 min. After the extraction, the cells were returned to the 37°C, 5% CO_2 incubator, and propidium iodide (PI) dye was added for

co-incubation after 24 h. The culture medium would be replaced after 15-30 min and the cell observed under a fluorescence microscope.

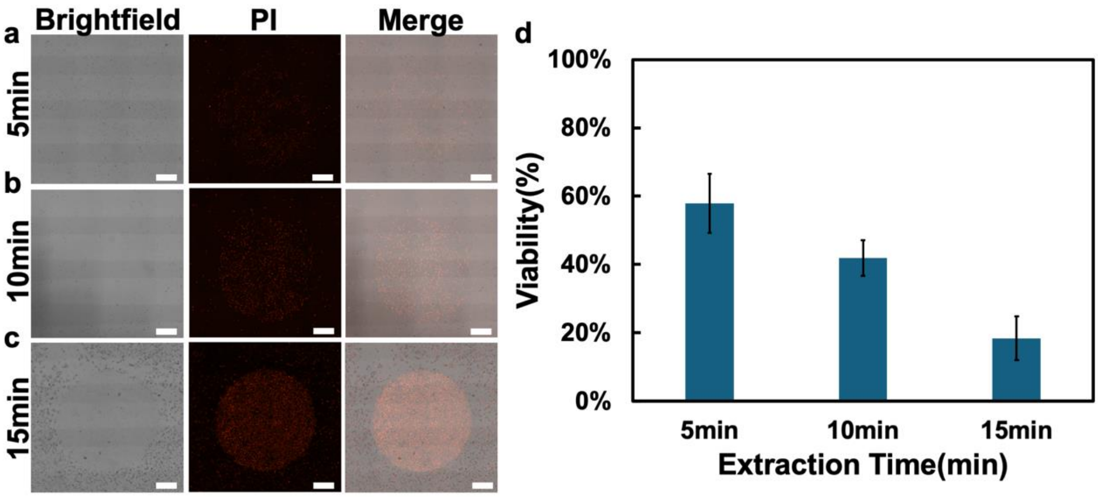

Figure S3. Optical and propidium iodide (PI) fluorescence (red) images of HeLa cells after 24 h incubation at different extraction times using a nanotube stamp of 1.0 μm, 2.2E7: a) 5 min, b) 10 min, and c) 15 min. d) HeLa cell viability after 24 h incubation with different extraction times using a nanotube stamp of 1.0 μm 2.2E7. Scale bar is 500 μm.

Using the same 15 min extraction time with different NT stamps of 1.0 μm, 3E6, 1.5 μm, 3E6, and 1.0 μm, 2.2E7 produce different results after 24 h incubation and staining with PI.

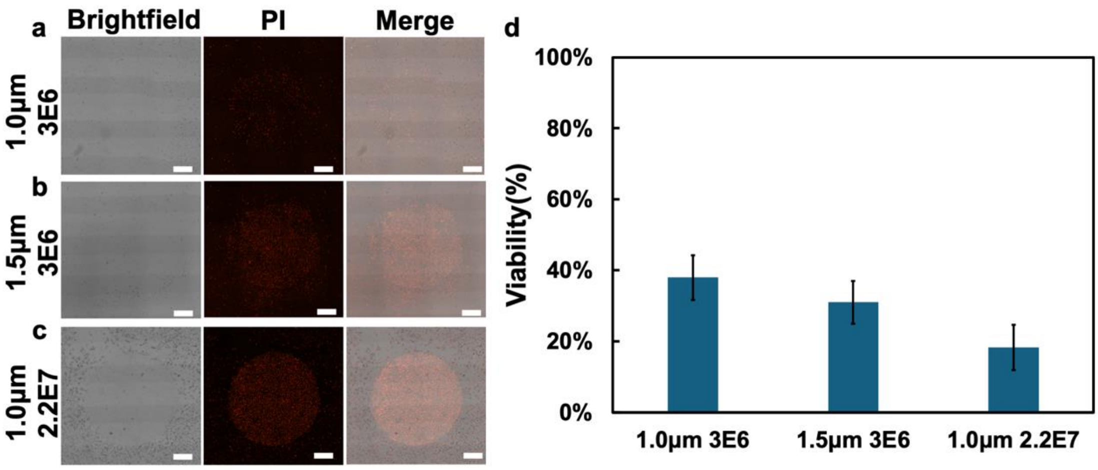

Figure S4. Optical and propidium iodide (PI) fluorescence (red) images of HeLa cells after 24 h incubation at 15 min extraction time with different nanotube stamps: a) 1.0 µm, 3E6, b) 1.5 µm, 3E6, and c) 1.0 µm, 2.2E7. d) HeLa cell viability after 24 h incubation with the different nanotube stamps at 15 min extraction time. Scale bar is 500 µm.

Efficiency of the transfer experiment

We assembled NT stamps of different glass tube diameters 6, 8, and 18 mm, using polydimethylsiloxane (PDMS) donuts to select the injection area and cultured HeLa cells inside. For extraction, we used a 6 mm glass tube with an 8 mm PDMS donut, an 8 mm glass tube with an 18 mm PDMS donut, and an 18 mm glass tube with an 18 mm PDMS donut for the selected injection area. This made the extracted part more obvious. For the injection, we used a 6 mm glass tube with a 4 mm PDMS donut, an 8 mm glass tube with a 6 mm PDMS donut, and an 18 mm glass tube with an 18 mm PDMS donut for the selected injection area. This enabled almost all cells within the injection area to be inserted.

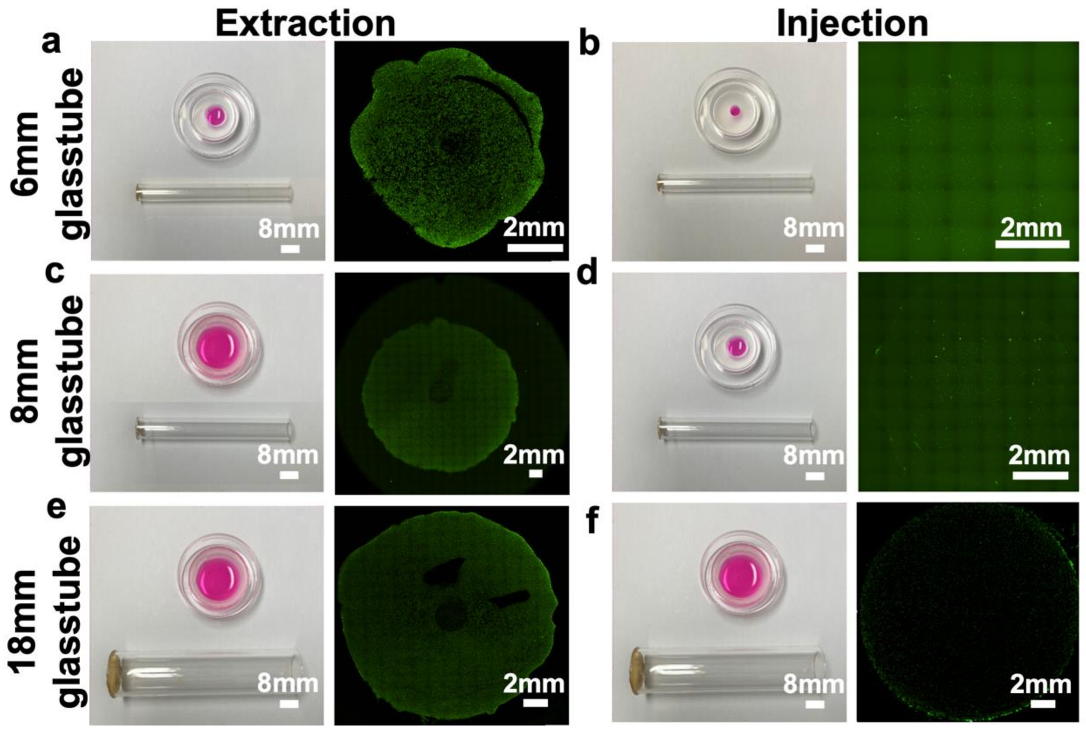

Figure S5. Images of cultured cells using polydimethylsiloxane (PDMS) donuts and nanotube stamps of diameters a, b) 6 mm, c, d) 8 mm, and e, f) 18 mm. Assembled image (left) and fluorescence image (right). Images of the 6 mm nanotube stamp: a) extraction, b) injection; images of the 8 mm nanotube stamp: c) extraction, d) injection, and images of the 18 mm nanotube stamp: e) extraction, f) injection.
